# P-262. Hydrogen Peroxide Fogging: Feasibility and CLABSI Impact in a Pediatric Hematology/Oncology Unit

**DOI:** 10.1093/ofid/ofae631.466

**Published:** 2025-01-29

**Authors:** Michael T Bigham, Jill Cirese, Jeffrey D Hord, Stephanie Williams, Beth A Carr, Hanan Haydar, Evelyn Pangonis, Tina Bair

**Affiliations:** Akron Children's Hospital, Akron, Ohio; Akron Children's Hospital, Akron, Ohio; Akron Children’s Hospital, Akron, Ohio; Akron Children's Hospital, Akron, Ohio; Akron Children's Hospital, Akron, Ohio; Akron children’s hospital, Brecksville, Ohio; Akron Childrens Hospital, Akron, Ohio; Akron Children's Hospital, Akron, Ohio

## Abstract

**Background:**

Hospital acquired infections, including central line infections (CLABSIs), increase morbidity and mortality in immunocompromised patients. Environmental decontamination with HHP fogging reduces bacterial growth and bioburden in the hospital setting. The investigators sought to test the feasibility of an oncology unit-based hybrid hydrogen peroxide (HHP) fogging program and assess the impact on CLABSIs.

**Methods:**

At a large Midwest freestanding children's hospital, this study evaluated the implementation of a HHP fogging program in a 20-room hematology/oncology inpatient unit and measured three endpoints:

1) the impact of fogging on aerobic colony counts (ACC) in a cohort of patient rooms cleaned using HHP Fogging (terminal clean + ultraviolet light + HHP fogging) compared to control rooms receiving standard cleaning (terminal clean + ultraviolet light);

2) the frequency, feasibility, and safety of fogging in a busy clinical unit; and

3) the important patient safety outcome, CLABSI rate, measured as rate per 1000 central line (CL) days of both mucosal-barrier infections (MBI) and non-MBI infections.

**Results:**

The pre-/post-cleaning ACC measured in the control rooms resulted in no decrease in average ACC while the HHP fogging intervention rooms demonstrated a 99.9% decrease in ACC. The observed fogging interval for each room did not deviate significantly from plan [plan = 19 days versus actual = 18.2 days (SD±4.7)]; with fogging scheduled for 2 days per week and with no unplanned patient room changes. There were no safety events associated with fogging. There was 100% chemical and biologic indicators passage rate affirming successful fogging cycle completion. Baseline CLABSI rate was 2.8 per 1000 CL days (non-MBI=1.3 per 1000 CL days). Since fogging began, there has been only 1 CLABSI, yielding a rate of 0.9 per 1000 CL days (non-MBI=0 per 1000 CL days). Days between CLABSI events averaged 35.4 days at baseline and only 1 MBI event since implementation (103 days after previous event).

**Conclusion:**

Hybrid hydrogen peroxide fogging is a safe, feasible, and effective method for reducing aerobic colony count in a busy hematology/oncology clinical unit and may reduce the CLABSI rate in an immunocompromised patient population.

**Disclosures:**

**Michael T. Bigham, MD, MBA**, ForTec Medical: Advisor/Consultant

